# Oral health among the elderly in 7 Latin American and Caribbean cities, 1999-2000: a cross-sectional study

**DOI:** 10.1186/s12903-015-0030-x

**Published:** 2015-04-09

**Authors:** Hema Singh, Rohan G Maharaj, Rahul Naidu

**Affiliations:** The University of the West Indies, St. Augustine, Trinidad and Tobago; Unit of Public Health and Primary Care, The Faculty of Medical Sciences, The University of the West Indies, St. Augustine, West Indies Trinidad and Tobago; Community Dentistry, The Faculty of Medical Sciences, The University of the West Indies, St. Augustine, Trinidad and Tobago

**Keywords:** Public health dentistry, Dental health survey, Elderly, Latin America, Caribbean

## Abstract

**Background:**

To describe the prevalence of missing teeth, use of bridges and dentures and unmet dental needs among those aged 60 years and above. The associations of these conditions with socio-demographics, type 2 diabetes mellitus and depression were also studied. The work was carried out in 7 Latin American and Caribbean (LAC) cities in 1999-2000.

**Methods:**

A secondary analysis was conducted on the Survey of Health and Well-Being of Elders (SABE) dataset. The 7 cities were Buenos Aires, Bridgetown, São Paulo, Santiago, Havana, Mexico City and Montevideo. This survey did not employ any oral examinations. Descriptive statistics, chi-square and regression analysis were used to test for associations.

**Results:**

Data for 10 902 persons were analyzed. Females made up 62% of the population. Across the SABE population, between 93.7% (Mexico City) to 99.9% (Santiago) reported missing teeth, with an average of 97.5%. Of those with missing teeth, between 55.1% (Mexico City) and 82.4% (São Paulo) reported having bridges or dentures, with an average of 70.1% across all SABE cities. The proportion of the SABE population with ‘unmet dental needs’ ranged from 85.8% (Santiago) to 98.4% (Havana), with an average of 94.5%. Bridgetown, São Paulo and Mexico City demonstrated a statistically significant association between aging and tooth loss. Generally a greater proportion of females (97.6%) reported tooth loss compared with males (96.8%), but in only São Paulo and Montevideo was there a statistically significant association between sex and tooth loss. Generally those with higher education reported less tooth loss, primary education (97.6% had tooth loss), secondary (96.8%) and tertiary (94.7%). All the SABE cities except Buenos Aires demonstrated a statistically significant association between tooth loss and education.

**Conclusions:**

The prevalence of missing teeth, use of bridges and dentures and unmet dental needs were high in the SABE cities in 1999-2000. In general across the SABE cities, the elderly with the most missing teeth were less educated or less likely to be a professional. They tended to be not working and were receiving a pension. Additionally they were less likely to report their health as ‘excellent’, were diabetic and were more likely to give responses suggestive of depression.

## Background

The Latin America and the Caribbean (LAC) population is ageing at unprecedented rates. The UN Database reports that by 2025 the elderly will increase by 300% in developing countries, especially in Latin America [1,2]. As life expectancy increases, so too does the desire for improved quality of life (QoL). QoL is dependent upon many factors including nutrition and enjoyment of food which involves adequate mastication and oral health [1].

Older people have more complex oral health needs. Oro-facial and dental pain or missing, discoloured and broken teeth can adversely affect people’s health, confidence and well-being. The resultant facial shape change which occurs may lead to an unwillingness to carry out everyday activities [1,3-5]. Many elderly also suffer from non-communicable diseases (NCDs) that can affect general and oral health. The required medications for these NCDs often cause reduced quantity and quality of saliva, thereby increasing the risk for tooth decay and other oral diseases [6]. Additionally, ill-fitting dentures affect patients’ QoL by making certain foods difficult to chew. Finally, oral cancer is also common in this age group and may develop after years of tobacco and alcohol abuse [7].

Whilst oral health care services are available in developed countries, utilisation is low among the elderly [8]. In low income countries where access to health care is poor especially in rural areas, elderly people experience high levels of oral health problems. In addition to socioeconomic factors, issues of limited availability and access to oral care make the elderly more vulnerable to developing oral diseases. The problem is further compounded in developing countries with diets rich in refined carbohydrates, and little allocation of health budgets to the prevention of oral diseases. Dental health resources cost developed countries 5-10% of health care expenditure per year [7] and oral disease is the fourth most expensive disease to treat [7]. Research in Latin America reveal that 60-70% of Mexicans over age 65 years have few or no teeth and gum disease and untreated caries are highly prevalent [7]. In South Brazil, poorer QoL are associated with depression and difficulty to chew food [9]. Aging populations therefore pose a significant challenge to healthcare systems. Appropriate oral health policies and strategies are needed to address these challenges.

This paper employed a secondary analysis of the Survey on Health, Well-Being, and Aging in Latin America and the Caribbean (SABE) dataset [10] to achieve the following objectives:

1. To describe the prevalence of oral health issues in the elderly population in 7 Latin American and Caribbean cities in 1999-2000.
2. To investigate associations between demographic variables, oral health and NCDs in this population.

Although dated, the information can provide a background of past oral health in preparation for future policies, strategies and research.

## Method

The Survey on Health, Well-Being, and Aging in Latin America and the Caribbean (SABE) [10] was a cross-sectional study conducted between October 1999 and December 2000. It set out to examine health (including oral health) conditions and limitations of persons aged 60 years and above, and living in private households. The surveys were undertaken in seven cities: Buenos Aires (Argentina), Bridgetown (Barbados), São Paulo (Brazil), Santiago (Chile), Havana (Cuba), Mexico City (Mexico) and Montevideo (Uruguay). SABE was funded by the Pan American Health Organisation (PAHO/WHO) [10]. The data base is to be used only for statistical reporting and analysis and is publicly available from: http://www.icpsr.umich.edu/icpsrweb/NACDA/studies/3546?archive=NACDA&q=SABE.

Demographic variables such as age, sex, race, education, birthplace, religion, ethnicity, marital status, and income were collected along with cognitive, health (including dental), functional and nutritional status, and use and accessibility of services. Dental health was measured by self-reporting rather than oral examination [11].

### Sampling target populations

The sampling target population from the SABE dataset were the sixty years (60) and older population living at home in urban areas of the respective cities [10].

### Sample design

Eligible participants were selected through a multistage clustered sample with stratification of the units. The sample was chosen in three selection stages of primary, secondary and tertiary sampling units; with two stages employed in Barbados and Brazil [10].

### Questionnaire

The SABE questionnaire was designed to produce information and to compare unique ageing processes in the LAC cities with other populations [10]. The modules extracted and included in this current paper are demographics, work history and income, self-reported overall health, oral health, diabetes and depression.

### SABE and Oral Health

The dentition aspects investigated in SABE are -

- ➢ The prevalence of oral disease in the elderly, ascertained from the question: Are you missing any teeth?
- ➢ Access to dental care: Do you have any bridges/dentures/false teeth?
- ➢ Unmet Dental Needs: In the SABE, the Geriatric Oral Health Assessment Index (GOHAI) scale was used to quantify the ’Unmet needs for oral health services’ of older adults [12,13]. If a participant had a score of 57 or less out of 60, they were regarded as having an ‘unmet dental need’.

Other information collected included self-reported overall assessment of health where responses ranged from ‘Excellent’ and ‘Very Good’ to ‘Bad’. Also collected was information on depression, the Yesavage Geriatric Depression Scale (GDS) Short form was used [14].

### Ethical issues

Ethical approval was granted for the conduct of the survey by the Pan American Health Organization Ethical Review Committee and the appropriate institutional review board in each city.

### Analysis

Descriptive statistics was used to compare proportions of affected elderly between the various cities and chi-square analysis was done to investigate whether any associations exist between demographic and disease variables and dentition in the elderly. Regression analysis for the ‘Unmet needs’ oral health variable against the demographic and disease variables was conducted to determine possible predictors of ‘Unmet dental health needs’ in the various cities. Statistical Package for the Social Sciences (SPSS) v. 12 was used. Statistical significance was set at *p* < 0.05.

## Results

The overall sample size of the SABE population was 10,902, females comprised 62%. The response rate ranged from 62.5% in Buenos Aires to 95.3% in Havana [10]. Across the SABE population, between 93.7% (Mexico City) to 99.9% (Santiago) reported missing teeth, with an average for all countries of 97.5%. See Tables 1, 2, 3, 4, 5, 6, 7, 8, 9, and 10.

**Table 1:** **Age and dentition for the SABE population**

**Row %**	**Are you missing any teeth?**			**Do you have bridges/dentures?**			**Unmet dental needs?**		
	**N (%)**			**N (%)**			**N (%)**		
**Age groups**	**Yes**	**No**	***p*** **value**	**Yes**	**No**	***p*** **value**	**Yes**	**No**	***p*** **value**
Bridgetown	N = 1474			N = 1417			N = 1477		
60 yrs and >	291 (96.4)	11 (3.6)	0.038	177 (61.9)	109 (38.1)	0.104	285 (94.1)	18 (5.9)	
61 - 65 yrs.	310 (97.8)	7 (2.2)		171 (56.4)	132 (44.6)		307 (96.2)	12 (3.8)	0.182
66 - 70 yrs.	298 (99.3)	2 (0.7)		180 (62.5)	108 (37.5)		276 (93.9)	18 (6.1)	
71 - 75 yrs.	248 (99.2)	2 (0.8)		161 (66.0)	83 (34.0)		230 (92.0)	20 (8.0)	
76 - 80 yrs.	175 (99.4)	1 (0.6)		95 (56.2)	74 (43.8)		163 (92.6)	13(7.4)	
81 yrs. and up	133 (98.5)	2 (1.5)		69 (54.3)	58 (45.7)		131 (97.0)	4 (3.0)	
Buenos Aires	N = 1027			N = 998			N = 1028		
60 yrs and >	46 (95.8)	2 (4.2)	0.441	30 (66.7)	15 (33.3)	0.164	47 (97.8)	1 (2.1)	0.423
61 - 65 yrs.	241 (98.0)	5 (2.0)		178 (73.6)	64 (26.4)		237 (96.3)	9 (3.7)	
66 - 70 yrs.	252 (98.4)	4 (1.6)		185 (73.4)	67 (26.6 )		243 (94.9)	13 (5.1)	
71 - 75 yrs.	233 (97.1)	7 (2.9)		176 (76.5)	54 (23.5)		235 (97.9)	5 (2.1)	
76 - 80 yrs.	134 (98.5)	2 (1.5)		97 (74.0)	34 (26.0)		134 (97.8)	3 (2.2)	
81 yrs. and up	101 (100)	0 (0.0)		79 (80.6)	19 (19.4)		96 (95.0)	5 (5.0)	
São Paulo	N = 2143			N = 2129			N = 2143		
60 yrs and >	76 (100)	0 (0.0)	0.045	66 (86.8)	10 (13.2)	0.022	73 (96.1)	3 (3.9)	0.747
61 - 65 yrs.	429 (98.8)	5 (1.2)		331 (77.2)	98 (22.8)		413 (95.2)	21 (4.8)	
66 - 70 yrs.	366 (99.2)	3 (0.8)		308 (84.2)	58 (15.8)		356 (96.5)	13 (3.5)	
71 - 75 yrs.	379 (98.7)	5 (1.3)		311 (82.3)	67 (17.7)		373 (97.1)	11 (2.9)	
76 - 80 yrs.	421 (100)	0 (0.0)		352 (83.6)	69 (16.4)		404 (96.0)	17 (4.0)	
81 yrs. and up	459 (100)	0 (0.0)		358 (78.0)	101 (22.0)		438 (95.6)	21 (4.6)	
Santiago	N = 1299			N = 1281			N = 1301		
60 yrs and >	63 (100)	0 (0.0)	0.365	41 (67.2)	20 (32.8)	0.017	55 (87.3)	8 (12.7)	0.108
61 - 65 yrs.	278 (100)	0 (0.0)		180 (65.7)	94 (34.3)		244 (87.1)	36 (12.9)	
66 - 70 yrs.	327 (100)	0 (0.0)		202 (72.5)	121 (27.5)		290 (88.7)	37 (11.3)	
71 - 75 yrs.	247 (100)	0 (0.0)		178 (72.7)	67 (27.3)		216 (87.4)	31 (12.6)	
76 - 80 yrs.	182 (100)	0 (0.0)		133 (73.9)	47 (26.1)		153 (84.1)	29 (15.9)	
81 yrs. and up	201 (99.5)	1 (0.5)		147 (74.2)	51 (25.8)		162 (80.2)	40 (19.8)	
Havana	N = 1905			N = 1877			N = 1905		
60 yrs and >	112 (99.1)	1 (0.9)	0.838	69 (61.6)	43 (38.4)	0.007	113 (100)	0 (0.0)	0.452
61 - 65 yrs.	438 (98.0)	9 (2.0)		293 (66.9)	145 (33.1)		440 (98.4)	7 (1.6)	
66 - 70 yrs.	389 (98.7)	5 (1.3)		285 (73.3)	104 (26.7)		384 (97.5)	10 (2.5)	
71 - 75 yrs.	326 (99.1)	3 (0.9)		226 (69.3)	100 (30.7)		321 (97.6)	8 (2.4)	
76 - 80 yrs.	251 (98.4)	4 (1.6)		192 (76.5)	59 (23.5)		252 (98.8)	3 (1.2)	
81 yrs. and up	361 (98.4)	6 (1.6)		270 (74.8)	91 (25.2)		329 (97.8)	8 (2.2)	
Mexico City	N = 1873			N = 1717			N = 1876		
60 yrs and >	628 (89.0)	78 (11.0)	0.000	339 (54.1)	288(45.9)	0.620	662 (93.5)	46 (6.5)	0.255
61 - 65 yrs.	337 (90.8)	34 (9.2)		171 (51.0)	164 (49.0)		352 (94.9)	19 (5.1)	
66 - 70 yrs.	280 (93.6)	19 (6.4)		155 (55.6)	124 (44.4)		282 (94.3)	17 (5.7)	
71 - 75 yrs.	201 (95.7)	9 (4.3)		109 (54.5)	91 (45.5)		190 (94.5)	20 (9.5)	
76 - 80 yrs.	150 (95.5)	7 (4.5)		89 (59.7)	60 (41.3)		150 (95.5)	7 (4.5)	
81 yrs. and up	127 (97.7)	3 (2.3)		71 (55.9)	56 (45.1)		120 (94.5)	7 (5.5)	
Montevideo	N = 1450			N = 1365			N = 1450		
60 yrs and >	64 (91.4)	6 (9.6)	0.166	52 (79.7)	13 (21.3)	0.078	66 (94.3)	4 (5.7)	0.259
61 - 65 yrs.	291 (92.7)	23 (7.2)		209 (72.8)	78 (27.2)		301 (95.9)	13 (4.1)	
66 - 70 yrs.	366 (94.8)	20 (5.2)		289 (79.2)	76 (20.8)		376 (97.4)	10 (2.6)	
71 - 75 yrs.	282 (96.2)	11 (3.8)		214 (76.4)	66 (23.6)		277 (94.5)	16 (5.5)	
76 - 80 yrs.	220 (96.5)	8 (3.5)		163 (75.1)	54 (24.9)		217 (95.2)	11 (4.8)	
81 yrs. and up	153 (96.2)	6 (3.8)		129 (84.9)	23 (15.1)		156 (98.1)	3 (1.9)

**Table 2:** **Gender and dentition for the SABE population**

**Row N %**	**Are you missing any teeth?**			**Do you have bridges/dentures?**			**Unmet dental needs?**		
	**N (%)**			**N (%)**			**N (%)**		
**Sex**	**Yes**	**No**	***p*** **value**	**Yes**	**No**	***p*** **value**	**Yes**	**No**	***p*** **value**
Bridgetown	N = 1474			N = 1417			N = 1477		
Male	887 (97.9)	13 (2.1)	0.349	515 (61.9)	356 (38.1)	0.299	843 (93.5)	59 (6.5)	0.104
Female	562( 98.6)	12 (1.4)		338 (59.1)	208 (41.9)		549 (95.5)	26 (4.5)	
Buenos Aires	N = 1027			N = 998			N = 1028		
Male	370 (97.9)	12 (2.1)	0.765	268 (73.2)	98 (26.8)	0.143	368 (97.4)	10 (2.6)	0.255
Female	637 (98.2)	8 (1.8)		477 (75.5)	155 (25.5)		624 (96.0)	26 (4.0)	
São Paulo	N = 2143			N = 2129			N = 2143		
Male	872 (99.0)	9 (1.0)	0.039	634 (72.7)	238 (27.3)	0.000	846 (96.0)	35 (4.0)	0.937
Female	1258 (99.7)	4 (0.3)		1092 (86.9	165 (13.1)		1211 (96.0)	51 (4.0)	
Santiago	N = 1299			N = 1281			N = 1301		
Male	445 (99.8)	1 (0.2)	0.167	236 (53.8)	203 (46.2)	0.000	376 (84.3)	70 (15.7)	0.180
Female	853 (100)	0 (0.0)		645 (76.7)	197 (23.3)		744 (87.0)	111 (13.0)	
Havana	N = 1905			N = 1877			N = 1905		
Male	700 (98.9)	8 (1.1)	0.343	454 (64.9)	246 (35.1)	0.000	698 (98.6)	10 (1.4)	0.239
Female	1177 (98.3)	20 (1.7)		881 (74.9)	296 (25.1)		1171 (97.8)	26 (2.2)	
Mexico City	N = 1873			N = 1717			N = 1876		
Male	465 (91.7)	42 (8.3)	0.789	216 (46.8)	246 (53.2)	0.000	481 (94.9)	26 (5.1)	0.172
Female	1258 (92.1)	108 (7.9)		718 (57.2)	537 (42.8)		1275 (93.1)	94 (6.9)	
Montevideo	N = 1450			N = 1365			N = 1450		
Male	490 (92.5)	40 (7.5)	0.001	318 (65.4)	168 (34.6)	0.000	514 (97.0)	16 (3.0)	0.175
Female	886 (96.3)	34 (3.7)		737 (83.8)	142 (16.2)		879 (95.4)	41 (4.5)

**Table 3:** **Education level and dentition for the SABE population**

**Row N (%)**	**Are you missing any teeth? N (%)**			**Do you have bridges/dentures? N (%)**			**Unmet dental needs? N (%)**		
**Education level**	**Yes**	**No**	***p*** **value**	**Yes**	**No**	***p*** **value**	**Yes**	**No**	***p*** **value**
Bridgetown	N = 1456			N = 1399			N = 1459		
Primary	1119 (98.7)	15 (1.3)	0.049	665 (60.9)	427 (39.1)	0.281	1071 (94.2)	66 (5.8)	0.843
Secondary	217 (96.4)	8 (1.6)		126 (59.4)	86 (41.6)		214 (95.1)	11 (4.9)	
Higher/oth	96 (99.0)	1 (1.0)		50 (52.6)	45 (47.4)		91 (93.8)	6 (6.2)	
Buenos Aires	N = 989			N = 960			N = 990		
Primary	690 (98.4)	11 (1.6)	0.209	505 (74.5)	173 (25.6)	0.301	677 (96.6)	24 (3.4)	0.838
Secondary	184 (97.4)	5 (2.6)		136 (73.5)	49 (26.5)		182 (95.8)	8 (4.2)	
Higher/oth	95 (96.0)	4 (4.0)		78 (80.4)	19 (19.6)		96 (97.0)	3 (3.0)	
São Paulo	N = 1600			N = 1586			N = 1600		
Primary	1347 (99.6)	6 (0.4)	0.001	1140 (84.7)	206 (15.3)	0.729	1293 (95.6)	60 (4.4)	0.622
Secondary	70 (97.2)	2 (2.8)		59 (84.3)	11 (15.7)		69 (95.8)	3 (4.2)	
Higher/oth	170 (97.1)	5 (2.9)		140 (82.4)	30 (17.6)		170 (96.1)	5 (3.9)	
Santiago	N = 1138			N = 1126			N = 1139		
Primary	90 (100)	0 (0.0)	*	62 (68.9)	28 (31.1)	0.936	77 (85.6)	13 (14.4)	0.877
Secondary	143 (100)	0 (0.0)		96 (67.6)	46 (32.4)		125 (87.4)	18 (12.6)	
Higher/oth	905 (100)	0 (0.0)		618 (69.1)	276 (31.9)		778 (85.9)	128 (14.1)	
Havana	N = 1901			N = 1873			N = 1901		
Yes	1793 (98.7)	23 (1.3)	0.001	1270 (70.8)	523 (29.2)	0.126	1782 (98.1)	34 (1.9)	0.751
No	80 (94.1)	5 (5.9)		63 (78.8)	17 (21.2)		83 (97.6)	2 (2.4)	
Mexico City	N = 1553			N = 1415			N = 1554		
Primary	1014 (92.1)	87 (7.9)	0.020	531 (52.5)	480 (47.5)	0.000	1033 (93.7)	69 (6.3)	0.276
Secondary	115 (95.0)	6 (5.0)		77 (67.0)	38 (33.0)		115 (95.0)	6 (5.0)	
Higher/oth	291 (87.9)	40(22.1)		210 (72.7)	79 (27.3)		303 (91.5)	28 (8.5)	
Montevideo	N = 1382			N = 1300			N = 1382		
Primary	859 (96.5)	31 (3.5)	0.000	656 (76.7)	199 (23.3)	0.239	858	32 (3.6)	0.886
Secondary	221 (94.8)	12 (5.2)		177 (81.6)	40 (19.4)		224	9 (3.9)	
Higher/oth	231 (89.2)	28(11.8)		172 (75.4)	56 (24.5)		248	11 (4.2)	

*Santiago has 100% missing teeth for this variable unable to determine an association.

Havana has no education level variable, so “Did you attend school - yes/no” was used.

**Table 4:** **Marital status and dentition for the SABE population**

**Row %**	**Are you missing any teeth?**			**Do you have bridges/dentures?**			**Unmet dental needs? N (%)**		
**Marital status**	**Yes**	**No**	***p*** **value**	**Yes**	**No**	***p*** **value**	**Yes**	**No**	***p*** **value**
Bridgetown	N = 1459			N = 1402			N = 1462		
Unmarried	292 (98.7)	4 (1.3)	0.373	186 (63.9)	105 (36.1)	0.368	284 (94.4)	17 (5.6)	
Married	499 (97.7)	12 (2.3)		292 (59.8)	196 (40.2)		488 (95.1)	25 (4.9)	
Widow/er	407 (99.3)	3 (0.7)		232 (58.1)	167 (41.9)		382 (93.2)	28 (6.8)	0.164
Separated	151 (98.1)	3 (1.9)		94 (63.9)	53 (36.1)		149 (96.1)	6 (3.9)	
Divorced	81 (97.6)	2 (2.4)		42 (54.5)	35 (45.5)		74 (89.2)	9 (10.8)	
Buenos Aires	N = 1025			N = 996			N = 1026		
Unmarried	54 (94.7)	3 (5.3)	*	46 (82.1)	10 (17.9)	0.212	56 (98.2)	1 (1.8)	
Married	432 (97.7)	10 (2.3)		315 (72.7)	118 (27.3)		426 (96.4)	16 (3.6)	
Widow/er	42 (98.6)	6 (1.4)		313 (76.3)	97 (23.7)		413 (96.5)	15 (3.5)	0.886
Separated	88 (98.9)	1 (1.1)		60 (69.0)	27 (31.0)		85 (95.5)	4 (4.5)	
Divorced	10 (100)	0 (0.0)		9 (90.0)	1 (10.0)		10 (100)	0 (0.0)	
São Paulo	N = 2142			N = 2128			N = 2142		
Unmarried	103 (100)	0 (0.0)	*	80 (77.7)	23 (22.7)	0.139	95 (92.2)	8 (7.8)	
Married	1112 (99.2)	9 (0.8)		899 (80.9)	12 (19.1)		1082 (95.5)	39 (3.5)	
Widow/er	757 (99.6)	3 (0.4)		626 (82.7)	131 (17.3)		728 (95.8)	32 (4.2)	0.035
Separated	135 (99.3)	1 (0.7)		101 (74.8)	34 (25.2)		132 (97.1)	4 (2.9)	
Divorced	22 (100)	0 (0.0)		20 (90.9)	2 (19.1)		19 (86.4)	3 (13.6)	
Santiago	N = 1284			N = 1266			N = 1286		
Unmarried	94 (98.9)	1 (1.1)	*	56 (61.5)	35 (39.5)	0.013	85 (89.5)	10 (10.5)	
Married	560 (100)	0 (0.0)		362 (65.3)	192 (34.7)		495 (88.4)	65 (11.6)	
Widow/er	458 (100)	0 (0.0)		334 (73.7)	119 (26.3)		379 (82.4)	81 (17.6)	0.015
Separated	166 (100)	0 (0.0)		113 (69.3)	50 (31.7)		147 (88.6)	19 (11.4)	
Divorced	5 (100)	0 (0.0)		5 (100)	0 (0.0)		3 (60.0)	2 (40.0)	
Havana	N = 1902			N = 1874			N = 1902		
Unmarried	61 (93.8)	4 (6.2)	*	31 (50.8)	30 (49.2)	0.001	64 (98.5)	1 (1.5)	
Married	700 (98.5)	11 (1.5)		484 (69.1)	216 (30.9)		702 (98.7)	9 (1.3)	
Widow/er	658 (98.9)	7 (1.1)		494 (75.1)	164 (24.9)		648 (97.4)	17 (2.6)	0.534
Separated	247 (99.6)	1 (0.4)		179 (72.5)	68 (27.5)		243 (98.0)	5 (2.0)	
Divorced	208 (97.7)	5 (2.3)		145 (69.7)	63 (30.3)		209 (98.1)	4 (1.9)	
Mexico City	N = 1868			N = 1712			N = 1871		
Unmarried	89 (94.7)	5 (5.3)	0.002	52 (58.4)	37 (41.6)	0.063	92	2 (2.1)	
Married	978 (91.1)	95 (8.9)		515 (52.9)	458 (47.1)		1010	64 (6.0	
Widow/er	457 (94.0)	29 (6.0)		259 (56.6)	199 (44.4)		452	36 (7.4)	0.291
Separated	170 (92.9)	13 (7.1)		86 (51.2)	82 (48.8)		169	14 (7.7)	
Divorced	24 (75.0)	8 (5.0)		19 (79.2)	5 (20.8)		29	3 (9.4)	
Montevideo	N = 1444			N = 1360			N = 1444		
Unmarried	49 (94.2)	3 (5.8)	0.288	28 (57.1)	21 (42.9)	0.000	49 (94.2)	3 (5.8)	
Married	659 (93.7)	44 (6.2)		495 (75.8)	158 (24.2)		673 (95.7)	30 (4.3)	0.456
Widow/er	511 (96.4)	19 (3.6)		416 (82.1)	91 (17.9)		515 (97.2)	15 (2.8)	
Separated	82 (96.5)	3 (3.5)		55 (67.9)	26 (32.1)		80 (94.1)	5 (5.9)	
Divorced	70 (94.6)	4 (5.4)		57 (81.4)	13 (18.6)		70 (94.6)	4 (5.4)	

*Buenos Aires, São Paulo, Santiago and Havana has many cells with N < 5 for this variable therefore unable to determine an association.

**Table 5:** **Past occupation and dentition for the SABE population**

**Row %**	**Are you missing any teeth?**			**Do you have bridges/dentures?**			**Unmet dental needs? N (%)**		
**Past occupation**	**Yes**	**No**	***p*** **value**	**Yes**	**No**	***p*** **value**	**Yes**	**No**	***p*** **value**
Bridgetown	N = 1404			N = 1348			N = 1407		
Professionals	108 (54.3)	91 (45.7)	0.079	108 (54.3)	91 (45.7)	0.129	197 (93.8)	13 (6.2)	0.166
Office workers	371 (59.1)	257 (41.9)		371 (59.1)	257 (40.9)		622 (95.4)	30 (4.6)	
Manual/unskill	325 (62.4)	196 (31.6)		325 (62.4)	196 (37.6)		506 (92.8)	39 (7.2)	
Buenos Aires	N = 925			N = 898			N = 883		
Professionals	129 (97.7)	3 (2.3)	0.810	99 (78.0)	28 (22.0)	0.345	84 (93.3)	6 (6.7)	0.288
Office workers	248 (98.4)	4 (1.6)		187 (74.8)	63 25.2)		243 (96.3)	9 (3.6)	
Manual/unskill	533 (98.5)	8 (1.5)		375 (72.0)	146 (28.0)		525 (97.0)	16 (3.0)	
São Paulo	N = 2000			N = 1986			N = 2000		
Professionals	141 (97.2)	4 (2.8)	0.003	125 (88.7)	16 (11.3)	0.002	139 (95.9)	6 (4.1)	0.985
Office workers	535 (99.3)	4 (0.7)		409 (76.6)	125 (23.4)		517 (95.9)	22 (4.1)	
Manual/unskill	1311 (99.6)	5 (0.4)		1075 (82.)	236 (18)		1260 (95.7)	56 (4.3)	
Santiago	N = 1053			N = 1038			N = 1055		
Professionals	77 (100)	0 (0.0)	*	58 (77.3)	17 (22.7)	0.211	67 (87.0)	10 (13.0)	0.700
Office workers	207 (100)	0 (0.0)		136 (67.3)	66 (32.7)		178 (85.2)	31 (14.8)	
Manual/unskill	769 (100)	0 (0.0)		514 (67.5)	247 (32.5)		672 (87.4)	97 (12.6)	
Havana	N = 1584			N = 1561			N = 1584		
Professionals	266 (96.4)	10 (3.6)	0.003	180 (67.7)	86 (32.3)	0.538	274 (99.3)	2 (0.7)	0.160
Office workers	568 (99.3)	4 (0.7)		405 (71.3)	163 (28.7)		558 (97.6)	14 (2.4)	
Manual/unskill	727 (98.8)	9 (1.2)		515 (70.8)	212 (29.2)		725 (98.5)	11 (1.5)	
Mexico City	N = 1532			N = 1410			N = 1533		
Professionals	113 (86.9)	17 (13.1)	0.035	85 (75.2)	28 (24.8)	0.000	119 (91.5)	11 (8.5)	0.323
Office workers	477 (93.7)	32 (6.3)		262 (55.3)	212 (44.7)		469 (92.1)	40 (7.9)	
Manual/unskill	823 (92.2)	70 (7.8)		404 (49.1)	419 (50.9)		840 (94.0)	54 (6.0)	
Montevideo	N = 1310			N = 1233			N = 1310		
Professionals	183 (89.3)	22 (10.7)	0.000	140 (78.2)	39 (21.8)	0.019	196 (4.4)	9 (95.5)	0.470
Office workers	304 (96.5)	11 (3.5)		248 (81.8)	55 (18.2)		306 (2.9)	9 (97.1)	
Manual/unskill	756 (95.7)	34 (4.3)		555 (73.9)	196 (26.1)		755 (4.4)	35 (95.5)	

*Santiago has 100% missing teeth for this variable therefore no cell for a p value.

**Table 6:** **Working status and dentition for the SABE population**

**Row %**	**Are you missing any teeth?**			**Do you have bridges/dentures?**			**Unmet dental needs? N (%)**		
**Working status**	**Yes**	**No**	***p*** **value**	**Yes**	**No**	***p*** **value**	**Yes**	**No**	***p*** **value**
Bridgetown	N = 1409			N = 1353			N = 1412		
Yes	247 (97.2)	7 (2.8)	0.191	138 (56.6)	106 (43.4)	0.255	243 (95.7)	11 (4.3)	0.267
No	137 (98.4)	18 (1.6)		671 (60.5)	438 (39.5)		1087 (93.9)	71 (6.1)	
Buenos Aires	N = 929			N = 902			N = 929		
Yes	246 (97.6)	6 (2.4)	0.258	186 (75.3)	61 (24.7)	0.508	239 (94.8)	13 (5.2)	0.138
No	668 (98.7)	9 (1.3)		479 (73.1)	176 (26.9)		656 (96.9)	21 (3.1)	
São Paulo	N = 2004			N = 1990			N = 2004		
Yes	432 (98.9)	5 (1.1)	0.145	344 (79.8)	87 (20.2)	0.476	422 (96.6)	15 (3.4)	0.371
No	1559 (99.5)	8 (0.5)		1268 (81.3)	291 (18.7)		1498 (95.6)	69 (4.4)	
Santiago							N = 1188		
Yes	313 (100)	0.0	*	191 (61.8)	118 (39.2)	0.007	274 (87.3)	40 (12.7)	0.659
No	873 (100)	0.0		604 (70.2)	257 (29.8)		754 (86.1)	120 (13.7)	
Havana	N = 1905			N = 1807			N = 1905		
Yes	346 (98.3)	6 (1.7)	0.685	236 (68.2)	110 (31.8)	0.185	346 (98.3)	6 (1.7)	0.777
No	1531 (98.6)	22 (1.4)		1099 (71.8)	432 (29.2)		1523 (98.1)	30 (1.9)	
Mexico City	N = 1569			N = 1443			N = 1571		
Yes	573 (88.7)	73 (11.3)	0.000	284 (49.6)	289 (51.4)	0.019	603	43 (6.7)	0.904
No	873 (94.6)	50 (5.4)		486 (55.9)	383 (44.1)		862	63 (6.8)	
Montevideo	N = 1327			N = 1250			N = 1327		
Yes	227 (91.9)	20 (8.1)	0.015	170 (75.2)	56 (24.8)	0.645	238	9 (3.6)	0.661
No	1023 (95.6)	47 (4.4)		785 (76.7)	239 (23.3)		1034	46 (4.3)

**Table 7:** **Pension and dentition for the SABE population**

**Row %**	**Are you missing any teeth?**			**Do you have bridges/dentures?**			**Unmet dental needs? N (%)**		
**Pension?**	**Yes**	**No**	***p*** **value**	**Yes**	**No**	***p*** **value**	**Yes**	**No**	***p*** **value**
Bridgetown	n = 1454			N = 1397			N = 1457		
Yes	995 (99.0)	10 (1.0)	0.003	581 (60.1)	385 (39.9)	0.884	944 (93.7)	63 (6.3)	0.229
No	435 (96.9)	14 (3.1)		261 (60.6)	170 (30.4)		429 (95.3)	21 (4.7)	
Buenos Aires	N = 1014			N = 902			N = 1014		
Yes	668 (98.2)	12 (1.8)	0.710	186 (75.3)	61 (24.7)	0.508	657 (96.5)	24 (3.5)	0.949
No	326 (97.9)	7 (2.1)		479 (73.1)	126 (26.9)		321 (96.4)	12 (3.6)	
São Paulo	N = 2135			N = 2121			N = 2135		
Yes	1689 (99.4)	10 (0.6)	0.812	1373 (81.3)	316 (18.7)	0.727	1631 (96.0)	68 (4.0)	0.905
No	433 (99.3)	3 (0.7)		348 (80.6)	84 (19.4)		418 (95.9)	18 (4.1)	
Santiago	N = 1297			N = 1279			N = 1299		
Yes	1284 (100)	0 (0.0)	0.000	868 (68.5)	399 (61.5)	0.085	1107 (86.1)	179 (13.9)	0.879
D/K	12 (92.3)	1 (7.7)		11 (91.7)	1 98.3)		11 (84.6)	2 (15.4)	
Havana	N = 1448			N = 1427			N = 1444		
Yes No	1427 (98.5)	21 (1.5)	*	1015 (71.1)	412 (28.9)	*	1421 (98.1)	27 (1.9)	*
Mexico City	N = 1852			N = 1696			N = 1854		
Yes	406 (93.1)	30 (6.9)	0.286	252 (62.5)	151 (37.5)	0.000	408 (93.6)	28 (6.4)	0.955
No	1296 (91.5)	120 (8.5)		667 (51.6)	626 (48.4)		1328 (93.7)	90 (6.3)	
Montevideo	N = 1445			N = 1361			N = 1445		
Yes	1108 (95.4)	54 (4.6)	0.155	859 (78.1)	241 (21.9)	0.117	1122 (96.6)	40 (3.4)	0.143
No	264 (93.3)	19 (6.7)		192 (73.6)	69 (26.4)		268 (94.7)	15 (5.3)	

*Havana has 100% elderly persons receiving pension therefore no cell for a p value.

**Table 8:** **Depression and Dentition for the SABE population**

**Row %**	**Are you missing any teeth? N (%)**			**Do you have bridges/dentures? N (%)**			**Unmet dental needs N (%)**		
**Depression**	**Yes**	**No**	***p*** **value**	**Yes**	**No**	***p*** **value**	**Yes**	**No**	***p*** **value**
Bridgetown	N = 1474			N = 1445			N = 1508		
No	1403 (98.3)	24 (1.7)	0.816	834 (60.4)	546 (39.6)	0.041	1358 (94.6)	81 (5.6)	0.571
Yes	46 (97.9)	1 (2.1)		31 (47.7)	34 (52.3)		64 (92.8)	5 (7.2)	
Buenos Aires	N = 1027			N = 1013			N = 1043		
No	916 (97.9)	20 (2.1)	0.159	673 (74.0)	236 (26.0)	0.833	903 (96.4)	34 (3.6)	0.352
Yes	91 (100)	0 (0.0)		76 (73.9)	28 (26.9)		104 (98.1)	2 (1.9)	
São Paulo	N = 2143			N = 2129			N = 2143		
No	1860 (99.4)	12 (0.6)	0.590	1503 (80.8)	356 (19.2)	0.495	1797 (96.0)	75 (4.0)	0.967
Yes	270 (99.6)	1 (0.4)		223 (82.6)	47 (17.4)		260 (95.9)	11 (4.1)	
Santiago	N = 1299			N = 1281			N = 1301		
No	1004 (99.1)	1 (0.1)	0.588	687 (69.5)	301 (30.5)	0.281	859 (85.3)	148 (14.7)	0.130
Yes	294 (100)	0 (0.0)		195(66.2)	99 (33.8)		261 (88.8)	33 (11.2)	
Havana	N = 1905			N = 1877			N = 1905		
No	1656 (98.6)	23 (1.4)	0.323	1185 (71.6)	471 (28.4)	0.256	1649 (98.2)	30 (1.8)	0.368
Yes	221 (97.8)	5 (2.2)		150 (67.9)	71 (32.1)		220 (97.3)	6 (2.7)	
Mexico City	N = 1873			N = 1717			N = 1817		
No	1377 (91.1)	135 (8.9)	0.003	784 (57.2)	587 (42.8)	0.000	1414 (93.3)	101 (6.7)	0.327
Yes	346 (95.8)	15 (4.2)		150 (43.4)	196 (56.6)		342 (94.7)	19 (5.3)	
Montevideo	N = 1450			N = 1365			N = 1450		
No	1229 (95.1)	64 (4.9)	0.445	949 (77.9)	269 (22.1)	0.112	1238 (95.7)	55 (4.3)	0.070
Yes	147 (93.6)	10 (6.4)		106 (72.1)	41 (27.9)		155 (98.7)	2 (1.3)

**Table 9:** **Diabetes and Dentition for the SABE population**

**Row %**	**Are you missing any teeth?**			**Do you have bridges/dentures?**			**Unmet dental needs? N (%)**		
**Diabetes**	**Yes**	**No**	***p*** **value**	**Yes**	**No**	***p*** **value**	**Yes**	**No**	***p*** **value**
Bridgetown	N = 1469			N = 1412			N = 1472		
Yes	315 (99.10	3 (0.9)	0.237	191 (62.4)	115 (37.6)	0.401	291 (90.9)	29 (9.1)	
No	1129 (98.1)	22 (1.9)		661 (59.8)	445 (40.2)		1096 (95.1)	56 (4.9)	0.004
Buenos Aires	N = 1023			N = 985			N = 1024		
Yes	128 (98.5)	2 (1.5)	0.713	494 (75.0)	165 (25.0)	0.649	125 (96.2)	5 (3.8)	0.827
No	875 (98.0)	18 (2.0)		240 (73.6)	86 (26.4)		863 (96.5)	31 (3.5)	
São Paulo	N = 2126			N = 2112			N = 2143		
Yes	377 (99.2)	3 (0.6)	0.840	304 (80.6)	73 (19.4)	0.060	370 (97.4)	10 (2.6)	0.210
No	1736 (99.4)	10 (0.4)		1412 (81.4)	323 (18.6)		1670 (95.6)	76 (4.4)	
Santiago	N = 1283			N = 1265			N = 1285		
Yes	173 (100)	0 (0.0)	0.693	126 (72.8)	47 (27.2)	0.224	153 (88.4)	20 (11.6)	0.333
No	1109 (99.9)	1 (0.1)		745 (68.2)	347 (31.8)		953 (85.7)	159 (14.3)	
Havana	N = 1903			N = 1876			N = 1899		
Yes	288 (99.3)	2 (0.7)	0.254	226 (78.5)	62 (21.5)	0.003	280 (96.6)	10 (3.4)	0.315
No	1588 (98.5)	25 (1.5)		1109 (69.8)	479 (30.2)		1587 (98.4)	126 (1.6)	
Mexico City	N = 1862			N = 1708			N = 1864		
Yes	347 (93.5)	24 (6.5)	0.224	171 (49.7)	173 (50.3)	0.045	346 (93.0)	26 (7.0)	0.628
No	1366 (91.6)	125 (8.4)		760 (55.7)	604 (44.3)		1398 (93.7)	94 (6.3)	
Montevideo	N = 1445			N = 1360			N = 1445		
Yes	175 (93.1)	13 (6.9)	0.232	134 (77.0)	40 (23.0)	0.888	182 (96.8)	6 (3.2)	0.569
No	1196 (95.1)	61 (4.9)		919 (77.5)	267 (22.5)		1206 (95.9)	51 (4.1)

**Table 10:** **Self-reported overall health and dentition for the SABE population**

**Row %**	**Are you missing any teeth?**			**Do you have bridges/dentures?**			**Unmet dental needs? N (%)**		
**Self reported health**	**Yes**	**No**	***p*** **value**	**Yes**	**No**	***p*** **value**	**Yes**	**No**	***p*** **value**
Bridgetown	N = 1474			N = 1445			N = 1508		
Excellent	56 (98.2)	1 (1.8)	0.218	33 (60.0)	22 (40.0)	0.391	57 (98.3)	1 (1.7)	0.368
Very good	163 (98.8)	2 (1.2)		89 (56.0)	70 (44.0)		164 (94.8)	9 (5.2)	
Good	501 (97.3)	14 (2.7)		289 (57.5)	214 (42.5)		496 (94.3)	30 (5.7)	
Fair	648 (98.8)	8 (1.2)		405 (62.3)	245 (37.7)		626 (93.4)	44 (6.6)	
Bad	81 (100)	0 (0.0)		49 (62.8)	29 (37.2)		79 (97.5)	2 (2.5)	
Buenos Aires	N = 995			N = 982			N = 1042		
Excellent	44 (97.8)	1 (2.2)	0.301	30 (71.4)	12 (28.6)	0.800	43 (93.5)	3 (6.5)	0.271
Very good	148 (100)	0 (0.0)		104 (73.2)	38 (26.8)		144 (97.3)	4 (2.7)	
Good	447 (97.6)	11 (2.4)		334 (74.4)	115 (25.6)		447 (96.3)	17 (3.7)	
Fair	284 (97.3)	8 (2.7)		220 (73.8)	78 (26.2)		294 (97.7)	7 (2.3)	
Bad	52 (100)	0 (0.0)		35 (68.6)	16 (31.4)		50 (96.2)	2 (3.8)	
São Paulo	N = 2143			N = 2129			N = 2143		
Excellent	90 (96.8)	3 (3.2)	0.001	79 (87.8)	11 (12.2)	0.171	90 (96.8)	3 (3.2)	0.927
Very good	128 (97.7)	3 (2.3)		107 (83.6)	21 (16.4)		125 (95.4)	6 (4.6)	
Good	730 (99.6)	3 (0.4)		596 (81.8)	133 (18.2)		702 (95.8)	31 (4.2)	
Fair	983 (99.7)	3 (0.3)		792 (80.6)	191 (19.4)		946 (95.9)	40 (4.1)	
Bad	199 (99.5)	1 (0.5)		152 (76.4)	47 (23.6)		194 (97.0)	6 (3.0)	
Santiago	N = 1299			N = 1281			N =1301		
Excellent	27 (100)	0 (0.0)	0.661	14 (51.9)	13 (48.1)	0.038	22 (81.5)	5 (18.5)	0.885
Very good	53 (100)	0 (0.0)		38 (77.6)	11 (22.3)		46 (86.8)	7 (13.2)	
Good	380 (99.7)	1 (0.3)		239 (64.6)	131 (35.4)		332 (87.1)	49 (12.9)	
Fair	559 (100)	0 (0.0)		397 (71.4)	159 (28.6)		483 (86.1)	78 (13.9)	
Bad	279 (100)	0 (0.00		103 (69.2)	86 (30.8)		237 (84.9)	42 (15.1)	
Havana	N = 1905			N = 1877			N = 1905		
Proxy	170 (96.6)	6 (3.4)	0.293	109 (64.1)	61 (32.9)	0.009	174 (98.9)	2 (1.1)	0.090
Excellent	33 (100)	0 (0.0)		19 (57.6)	14 (42.4)		32 (97.0)	1 (3.0)	
Very good	57 (100)	0 (0.0)		41 (71.9)	16 (28.1)		57 (100)	0 (0.0)	
Good	547 (98.7)	7 (1.3)		388 (70.9)	159 (29.1)		548 (98.9)	6 (1.1)	
Fair	850 (98.6)	12 (1.4)		634 (74.6)	216 (25.4)		844 (97.9)	18 (2.1)	
Bad	220 (98.7)	3 (1.3)		144 (65.5)	76 (34.5)		214 (96.0)	9 (4.0)	
Mexico City	N =1868			N =1713			N = 1871		
Excellent	42 (89.4)	5 (10.6)	0.000	33 (78.6)	9 (21.4)	0.002	43 (91.7)	4 (8.3)	0.728
Very good	67 (83.8)	13 (16.2)		43 (64.2)	24 (35.8)		77 (96.2)	3 (3.8)	
Good	395 (89.2)	48 (10.8)		228 (57.7)	167 (42.3)		415 993.7)	28 (6.3)	
Fair	900 (92.7)	71 (7.3)		466 (52.0)	431 (48.0)		907 (93.1)	67 (6.9)	
Bad	314 (96.0)	13 (4.0)		163 (52.2)	149 (47.8)		309 (94.5)	18 (5.5)	
Montevideo	N = 1450			N = 1365			N = 1450		
Excellent	84 (84.8)	15 (15.2)	0.000	71 (84.5)	12 (15.5)	0.068	96	3 (3.0)	0.626
Very good	143 (89.9)	16 (10.1)		105 (75.5)	36 (24.5)		152	7 (4.4)	
Good	663 (96.10	26 (3.9)		495 (78.6)	135 (21.4)		631	28 (4.2)	
Fair	426 (97.3)	12 (2.7)		324 (76.6)	99 (23.4)		420	18 (4.1)	
Bad	90 (94.7)	5 (5.3)		60 (68.2)	28 (31.8)		94	1 (1.1)	

### Socio-demographics (Age, education, marital status, occupational status) and prevalence of missing teeth

Across the SABE cities 2.5% of the population aged 60 years and above reported no missing teeth. A notable exception was the 60-65 years age groups in Mexico City and Montevideo where 8-11% reported having complete dentition. Bridgetown, São Paulo and Mexico City demonstrated a statistically significant association between aging and tooth loss. In all cities except Havana (M:F = 1.01:1), a greater proportion of females (97.6%) reported tooth loss compared with males (96.8%). In only São Paulo and Montevideo was there a statistically significant association between sex and tooth loss.

Generally those with higher education reported less tooth loss, among those with primary education, 97.6% reported tooth loss, secondary (96.8%) and tertiary (94.7%). All the SABE cities except Buenos Aires demonstrated a statistically significant association between tooth loss and education.

Greater proportions of manual and unskilled (92.5%), service workers and office employees (92.3%) reported having missing teeth compared with professionals (88.8%). São Paulo, Havana, Mexico City and Montevideo all demonstrated a statistically significant association between tooth loss and past employment. Across all SABE cities, among those with missing teeth, there were higher proportions currently not working (97.9%) than currently working (96.1%). Whilst among those with no missing teeth, there were more persons working (3.9%) than not (2.1%). This achieved statistical significance in Mexico City and Montevideo. Across the entire SABE population, among those with missing teeth, greater proportions were receiving a pension (97.5%) than not (95.2%); and among those with no missing teeth, greater proportions were not receiving a pension (4.8%) than those who were receiving a pension (2.5%). This achieved statistical significance in Bridgetown.

Throughout the SABE cities, all the categories of marital status report tooth loss range from 75% - 100%. With an average of 98.1%, Havana and Mexico City demonstrated a significant statistical association between tooth loss and marital status.

### Health conditions (depression and diabetes), self-reported overall health and prevalence of missing teeth

For all SABE cities except Montevideo, among those with missing teeth there were greater levels of depression (average 11.2%) compared with those not missing teeth (average 6.5%). This achieved statistical significance in Mexico City.

For all SABE cities except São Paulo and Montevideo, among those with missing teeth there were greater levels of diabetes (average = 16.5%) compared with those not missing teeth (average = 12.3%). This did not achieve statistical significance in any of the SABE cities.

Among those with missing teeth, 0.3% (Mexico City) to 6% (Montevideo) reported ‘Excellent’ health, with an average of 3.5% across 6 cities (excluding Havana which had different descriptors on the Likert scale). Among those with no missing teeth, between 0% (Montevideo) and 23.1% (São Paulo) reported ‘Excellent’ health, with an average of 9.3%. The association between ‘self-reported’ overall health and missing teeth achieved statistical significance for São Paulo, Mexico City and Montevideo.

### Socio-demographics and reporting bridges or dentures

Of those with missing teeth, between 55.1% (Mexico City) and 82.4% (São Paulo) reported having bridges or dentures, with an average of 70.1%.

São Paulo, Santiago and Havana all demonstrated a statistically significant association between aging and reporting the use of dentures or bridges. On average more females (73.4%) have bridges and dentures than males (62.7%), except in Bridgetown where it is the reverse. São Paulo, Santiago, Havana, Mexico City and Montevideo all demonstrated a statistically significant association between sex and reporting the use of dentures or bridges. The proportions of those with bridges/dentures was distributed on average equally among those with lower (70.1%) versus those with higher educational achievement (72.1%) across all SABE cities except in Mexico City. Here there was a statistically significant association between educational achievement and reporting the use of bridges or dentures.

Santiago, Havana and Montevideo all demonstrated a statistically significant association between marital status and reporting the use of dentures or bridges.

Generally larger proportions of professionals (74.2%) reported bridges or dentures compared with office or manual and unskilled workers (68.9%). In São Paulo, Mexico City and Montevideo there was a statistically significant association between occupation and reporting the use of bridges or dentures. Among those reporting using bridges or dentures there were no consistent pattern of current employment, except in Santiago and Mexico City. In these 2 cities there was a statistically significant association with greater proportions of those wearing dentures ‘not currently working’.

In 4 of the SABE cities among those reporting having bridges or dentures there were higher proportions receiving pensions. Only in Mexico City was there a statistically significant association.

### Health conditions and reporting bridges or dentures

In the SABE cities among those with missing teeth and reported wearing bridges or dentures 12.3% were ascertained to be depressed compared with 15.7% among those not wearing bridges or dentures. In Bridgetown and Mexico City this association achieved statistical significance.

Among the elderly with missing teeth and reporting use of bridges and dentures the proportion with diabetes was 17.9%, compared with those without bridges and dentures, diabetes was present in 20.7%. In Havana and Mexico City there was a statistically significant association among those wearing bridges and dentures and the presence of diabetes.

### Self-reported overall health

Among the SABE cities there was a consistent pattern of self-reported overall health and whether the respondents used bridges or dentures. In Santiago, Havana and Mexico City this achieved a statistically significant association.

### Socio-demographics and unmet oral health needs

The proportion of the SABE population with ‘unmet dental needs’ ranged from 85.8% (Santiago) to 98.4% (Havana), with an average of 94.5%. There were no statistically significant associations between unmet dental needs and age, sex, past occupation, education achievement, working status, or pension status. There were no statistically significant associations between marital status and unmet dental needs except in São Paulo and Santiago.

### Health conditions and unmet dental needs

There were no statistically significant associations between unmet dental needs and depression or self-reported health in any of the SABE cities. Similarly, there were no statistically significant associations between unmet dental needs and diabetes except for Bridgetown where more of those without diabetes have unmet dental needs. Regression analysis was conducted for each SABE city to determine which independent variables predicted having an ‘Unmet dental need’. There were no such independent variables identified except in Bridgetown where the ‘absence of diabetes’ predicted having an Unmet dental need. See Table 11.

**Table 11:** **Results of logistic regression to determine the independent variables associated with Unmet Oral health needs in the SABE population for Bridgetown**

**Bridgetown**	**B**	**S.E.**	**Wald**	**df**	**95% CI**	
					**Lower**	**Upper**
Age	.204	.247	.680	1	-.281	.689
Sex	-.399	.255	2.452	1	-.899	.100
Marital status	.195	.302	.418	1	-.397	.788
Education level	-.068	.316	.047	1	-.688	.552
Past occupation	.230	.352	.427	1	-.460	.920
Current working status	-.237	.369	.412	1	-.961	.487
Pension	.250	.279	.803	1	-.297	.797
Depression	.270	.478	.318	1	-.667	1.207
Diabetes	.583	.254	5.267	1	.085	1.081
Self-reported overall health	-12.307	.729	284.816	1	-13.736	-10.877
Constant	-15.598	.563	767.698	1	-16.701	-14.494

## Discussion

There has been a wealth of information arising from the SABE dataset, [10,12] but this paper is the first to describe the oral health of the population. Across the SABE population, in 1999-2000, 97.5% reported missing teeth, and of those with missing teeth, an average of 70.1% reported having bridges or dentures. Further, 94.5% were determined to have ‘unmet dental needs’, expressing difficulties with chewing, oral pain, speech and appearance, among other issues. Further analysis revealed associations with the presence of missing teeth and educational achievement or past employment across many, but not all the SABE cities.

### Social determinants of missing teeth

#### Education and Past occupation

Generally in this study those with higher education and those self-reporting their occupation as professionals reported less tooth loss. Those with a primary and secondary education had more tooth loss versus those with a tertiary education. All the SABE cities except Buenos Aires demonstrated a statistically significant association between tooth loss and education. Similarly, the manual and unskilled, service workers and office employees generally reported having more missing teeth compared with professionals. São Paulo, Havana, Mexico City and Montevideo all demonstrated a statistically significant association between tooth loss and past employment. This association with education is consistent with reports from the United States (US). In the National Health and Nutrition Examination Survey (NHANES) study, 23% of those with 0-8 years of education reported pain in biting or chewing compared with 10% of those with 13 or more years of education [15].

### Other surveys of dental disease in the Americas

Successive surveys of seniors over the age of 65 years in the US has shown that overall, the prevalence of tooth loss in seniors has decreased from the 1970 until the 2000s [16]. A more recent paper from 2005-8 reported that in this population 19.9% had untreated dental caries and almost 23% of were edentulous [17]. The data from this paper cannot be compared with these results however as different oral parameters were measured. In Latin America, a 2012 report of Decayed, Missing, Filled Teeth (DMFT) index showed a mean DMFT of 21.57 in the 65–74 years group [18]. Factors related to tooth loss in the 65–74 year-old group were education level *<*12 years (OR 2.54) and personal income (OR 1.66). This current paper has similar findings with respect to education. Two other South American countries have carried out national surveys including an oral examination in adults: Colombia [19] with a DMFT of 19.6 in the “older than 55” group and Brazil with a mean DMFT of 27.8 for the 65–74-year-old group in 2003 and a DMFT of 27.5 for the 65–74 years adults in 2010 [20]. However both these report DMFT, which cannot be compared directly with these results. Nevertheless they suggest, as does this paper, high levels of caries prevalence in the elderly.

### Depression and dentition

Depression has been well linked to dentition [21]. In Santiago, this association was found for the 35-44 age group but not the 65-74 year olds [18]. Similarly, in this current paper, no association was found except in Mexico City where those with missing teeth had twice the rate of depression than those with no missing teeth (8.9% vs. 4.2%, *p* < 0.003). In general, there were more depressed among those not wearing dentures (15.7%) than among those wearing dentures (12.3%).

This provides interesting areas for debate, including whether many of those elderly with missing teeth are generally less accepting of their loss and have higher rates of depression; and whether those without bridges and dentures, either had no access to care or cannot afford the services and have the resultant increased depression rates. These are areas for future study.

### Diabetes and oral health

Recent research have widened our understanding of the relationship between oral health and diabetes [22,23]. For example, periodontal disease has been shown to be is a strong predictor of mortality from ischemic heart disease (IHD) and diabetic nephropathy among Pima Indians with type 2 diabetes (T2DM) [24]. Also individuals with poorly controlled diabetes mellitus had a significantly higher prevalence of severe periodontitis than those without diabetes [25]. Tooth loss is considered the end point for untreated periodontal disease. The prevalence of periodontal disease is increasing in most aging societies suggesting it is a public health problem [26]. One study from Germany reported that the association between T2DM and tooth loss was statistically significant only for females [27]. In this current paper we studied the relationship between the presence of diabetes and oral health. We could demonstrate no relationship between those with diabetes and those reporting missing teeth. In 5 of the 7 SABE cities we could not demonstrate a relationship between diabetes and those reporting the use of bridges or dentures, the two exceptions being Havana and Mexico City. In both these cities those with diabetes made up about 17% of the users of bridges and dentures.

### Pension systems in SABE cities and relationship with oral health

Across the entire SABE population, among those with missing teeth, greater proportions were receiving a pension than not. The question for future study is whether those with less missing teeth are more educated and more healthy and therefore continue to work. Further, are they less likely to be receiving a pension, more engaged in life, and are subsequently less depressed? The findings of this paper appears to suggest that this is the case. The pension systems across the SABE cities are varied but generally universal with pensions available between age 60-65 years [28].

### Limitations

There are several limitations to this study, for example, data used in the SABE study is self-reported and not actual clinical examinations. This makes the comparison of this 1999-2000 data impossible with the 3 reported surveys of the DMFT index from Latin America. The SABE surveys were conducted in urban cities, whilst the more recent Latin American surveys report using nationally representative samples. This data was also collected fourteen years ago. Therefore, one can question how comparable or generalizable is it today. Nevertheless, the information does provide a baseline for other LAC cities and countries which have not conducted any subsequent surveys.

Additionally, the SABE study used self-perceived oral health which reflects people’s subjective and objective assessments of their oral health, and is highly associated with perceptions of treatment need and subsequent demand for dental services [11]. Future studies in these populations should use an oral examination to confirm participants’ perceptions.

### What’s next

This paper provides baselines which future studies can re-assess for change. These include the very high levels of missing teeth among all age intervals of those over 60 years, the high levels of unmet dental needs and the relatively high proportion of those requiring dental prostheses. The very high levels of ‘unmet dental needs’ across all the SABE cities is telling and future studies should evaluate how well this construct remains elevated as new dental interventions are introduced. There are also opportunities for extensive comparison of DMFT data across more LAC countries, with a focus on the elderly. As we saw above these are now available for 3 Latin American countries. This can assist in evaluating the different dental care models in LAC. Notably, the free health system of Havana did not particularly stand out as exemplary. Throughout the LAC there has been an epidemic of NCDs with diabetes being at the forefront. The evidence suggests that better periodontal care assists in better diabetes control [23]. In this study we could not demonstrate a link between diabetes and missing teeth. Future research in the LAC should investigate the cost-effectiveness of improving dental services to assist in combating the diabetes epidemic.

## Conclusions

The results of this secondary analysis illustrates that in 1999-2000, there was a high prevalence of missing teeth, bridge and dentures use and poorly met dental needs among the elderly in the 7 SABE cities of Latin America and the Caribbean.

In general across the SABE cities, the larger proportion of elderly reporting missing teeth were less educated or less likely to be a professional. They were also currently not working and were receiving a pension. Finally they were less likely to report their health as ‘excellent’, were diabetic and were more likely to give responses suggestive of depression.

## Abbreviations

DMFT: Decayed, Missing, Filled Teeth index
GDS: Geriatric Depression Scale
GOHAI: Geriatric Oral Health Assessment Index
IHD: Ischemic heart disease
LAC: Latin American and Caribbean
MPH: Master of Public Health
NHANES: National Health and Nutrition Examination Survey
NCD: Non-communicable disease
OR: Odds Ratio
PAHO: Pan American Health Organisation (PAHO)
QoL: Quality of life
SABE: Survey of Health and Well-Being of Elders
SPSS: Statistical Package for the Social Sciences
T2DM: Type 2 diabetes
US: United States
WHO: World Health Organization
